# Low density lipoprotein receptor-related protein 5 gene polymorphisms and osteoporosis in Thai menopausal women

**DOI:** 10.1186/s12952-016-0059-7

**Published:** 2016-09-01

**Authors:** Anong Kitjaroentham, Hathairad Hananantachai, Benjaluck Phonrat, Sangchai Preutthipan, Rungsunn Tungtrongchitr

**Affiliations:** 1 Department of Tropical Nutrition and Food Science, Faculty of Tropical Medicine, Mahidol University, Bangkok, Thailand; 2 Department of Social and Environmental Medicine, Faculty of Tropical Medicine, Mahidol University, Bangkok, Thailand; 3 Department of Clinical Tropical Medicine, Faculty of Tropical Medicine, Mahidol University, Bangkok, Thailand; 4 Department of Obstetrics and Gynecology, Faculty of Medicine, Ramathibodi Hospital, Mahidol University, Bangkok, Thailand

**Keywords:** Osteoporosis, Osteopenia, Low density lipoprotein receptor-related protein 5 (LRP5), Menopausal Thai women, Coding single nucleotide polymorphisms

## Abstract

**Background:**

Osteoporosis, characterized by low bone mineral density (BMD) and high bone fracture risk, is prevalent in Thai menopausal women. Genetic factors are known to play a key role in BMD. Low density lipoprotein receptor-related protein 5 (LRP5), a co-receptor in the Wnt/beta-catenin pathway, is involved in many aspects of bone biology. As coding single nucleotide polymorphisms (cSNPs) of *LRP5*, including A1330V (rs3736228), and Asian-related Q89R (rs41494349) and N740N (rs2306862), are associated with lowered BMD, this study aimed to determine the relationship between these *LRP5* polymorphisms and BMD in 277 Thai menopausal women.

**Results:**

Only rs3736228 deviated from the Hardy–Weinberg equilibrium of allele frequency (*p* = 0.022). The median, range and *p* value for the BMD related to each SNP parameter were compared (Mann–Whitney U test). Significant differences were observed between wild-type and risk alleles for both rs3736228 (total radial, *p* = 0.011; and radial 33, *p* = 0.001) and rs2306862 (radial 33: *p* = 0.015) SNPs, with no significant difference for rs41494349 SNP. Linkage disequilibrium was strong for both rs3736228 and rs2306862 SNPs. Haplotype analysis identified high CC frequency in both normal and osteopenia/osteoporosis groups, with a significant odds ratio for carrying the TT haplotype; however, this was non-significant after adjusting for age. Multivariate binary logistic regression analysis performed for rs3736228 showed that individuals with a body mass index <25 kg/m^2^ had an increased risk of osteoporosis for each decade, but the polymorphism had no effect.

**Conclusions:**

This study did not identify *LRP5* polymorphisms as a risk factor for osteoporosis in Thai menopausal women. Further studies with larger sample sizes are needed to further clarify the role of LRP5 as a genetic determinant of osteoporosis.

**Electronic supplementary material:**

The online version of this article (doi:10.1186/s12952-016-0059-7) contains supplementary material, which is available to authorized users.

## Background

Osteoporosis, a common metabolic bone disease, is characterized by low bone mineral density (BMD) and high risk of bone fracture [1]. During 40–50 years of age, women tend to lose bone mass significantly during the menopausal period because of greater bone resorption. In Thailand, the prevalence of osteoporosis in the lumbar spine and femoral neck of women aged 40–80 years is 19.8 % and 13.6 %, respectively. These figures are less than 5 % in women under 50 years of age, but increase more than 50 % in those older than 70 years of age [2]. Among factors which have an influence on osteoporosis, genetic factors play an essential role in bone density regulation. To date, individual genome-wide association studies (GWASs) and meta-analyses of GWASs have discovered more than 70 genes/loci to be associated with BMD phenotypes [3], particularly *ZBTB40*, *GPR177*, *FGFRL1*, *MEF2C*, *ESR1*, *WNT16*, *TNFRSF11B*, *SOX6*, *LRP5*, *AKAP11*, and *FOXL1* [4, 5]. Further, candidate gene association studies have suggested additional loci, e.g., *VDR* [6], *COL1A1* [7], *TGFB1* [8] and *PTH* [9].

The Wnt signaling pathway has an essential role in the regulation of many cellular processes, such as cell growth, differentiation, migration, polarity and apoptosis. In addition, the canonical Wnt/beta-catenin pathway is particularly involved with bone biology [10]. The transmembrane protein, low density lipoprotein receptor-related protein 5 (LRP5), acts as a co-receptor for the Wnt signaling pathway and it is widely expressed in many tissues including bone. In bone, it expresses by osteoblasts of the endoosteal and trabecular bone surface [11]. Analysis of two monogenic disorders having extreme bone phenotypes reveals that *LRP5* as a modulator of bone metabolism. Osteoporosis-pseudoglioma (OPPG), an autosomal recessive disease characterized by bone thinning and blindness due to abnormal eye development has been linked to loss of function mutation in *LRP5* [12]. OPPG-causing frameshift or nonsense mutations in *LRP5* extracellular domain have been identified and mutant LRP5 protein can be synthesized but not secreted. Dominant negative secreted form of LRP5 can reduce bone thickness in mouse calvarial explants [12]. In contrast to OPPG, an autosomal dominant high bone mass (HBM) traits results from a gain of function mutation in *LRP5*. Genetic studies of two unrelated families with increased BMD (spinal Z scores ≥ 5) identify a single missense mutation of *LRP5* resulting in amino acid substitution of a conserved glycine by valine at residue 171 (G171V) [13, 14]. Serum markers of affected individuals show increased bone formation but normal bone resorption [13]. Level of known target of Wnt signaling, the extracellular matrix protein fibronectin is also increased. Cell expressing LRP5_V171_ can abolish action of endogenous Wnt inhibitor, Dkk-1 resulting in increased LRP5 function and excessive bone formation [13]. Mice carrying both types of mutations have high and low bone mass phenotypes resembled that of human [11, 15]. Previous qualitative trait locus (QTL) work suggests that chromosome 11 q12–13 region where LRP5 located, may have important role in general BMD variance [16]. A cohort of British Caucasian adults covering broad range BMD studied by Koay et al demonstrates that *LRP5* polymorphisms is a genetic determinant of normal BMD variation [17].

Several coding single nucleotide polymorphisms (cSNPs) of *LRP5* have been reported. The most frequently studied and likely functional polymorphism of *LRP5*, A1330V (rs3736228) in exon 18, has been shown to affect BMD and increase fracture risk in various groups, including postmenopausal women [18–21]. It locates in LDL-repeat domain of LRP5 protein which may be responsible for mediating the receptor-ligand interaction [22]. HEK293T cells containing the LRP5-V1330 coexpressed with Wnt3a has reduced transcriptional activity measuring by TCF-Lef reporter assay as compared to wild type allele implying the functional significance of A1330V variant [19]. Another two *LRP5* cSNPs, Q89R (rs41494349) in exon 2 and N740N (rs2306862) in exon 10 are chosen because of their association with low BMD in Asian population [23–25]. Q89R is localized on the first of four beta-propellers, while N740N is on the third one [26]. Structural analysis of mutation occurred in the first propeller domain of *LRP5* suggest changing in local hydrophobic environment and subsequently possibly affecting interaction of LRP5 with other proteins [14]. Although the function of each LRP5 protein domain is still uncertain, these 3 cSNPs could have influence on LRP5 biological function and BMD. Therefore, *LRP5* genetic variations may have an effect on osteoporosis within the Thai population. Thus, we studied the relationship of *LRP5* polymorphisms (rs3736228, rs41494349 and rs2306862) and BMD in Thai menopausal women.

## Results

Basic characteristics, including age, body mass index (BMI) and BMD of menopausal women, are shown in Table 1. Among the genotype distribution of *LRP5* polymorphisms studied, those of rs41494349 and rs2306862 did not deviated significantly from Hardy–Weinberg Equilibrium (HWE), which is in contrast with rs3736228 (*p* = 0.022) (Table 2). Neither rs41494349 nor rs2306862 SNP was associated with risk of osteopenia/osteoporosis in terms of dominant or recessive model (Table 3). However, the genetic model of dominance for rs3736228 was assumed due to none of its homozygous (T/T) variant present.

**Table 1:** Characteristic data of the studied population

Characteristic	Median (Min–Max)	Number
Age (years)	57 (45–75)	277
Body mass index (BMI) (kg/m^2^)	22.79 (15.46–39.54)	277
Lumbar spine BMD (g/cm^2^)	1.013 (0.700–1.564)	205
Femoral neck BMD (g/cm^2^)	0.788 (0.434–1.224)	233
Total radial BMD (g/cm^2^)	0.477 (0.262–0.961)	277
Radial 33 BMD (g/cm^2^)	0.626 (0.315–0.818)	277
Total hip BMD (g/cm^2^)	0.871 (0.229–1.322)	233

BMD = bone mineral density

**Table 2:** Genotype distributions in subjects

Genotype	All subjects (N)	Allele frequency	HWE (*p*-value)
rs3736228			
CC	210	C = 0.88	0.022
CT	67	T = 0.12	
TT	0		
rs41494349			
AA	245	A = 0.94	0.239
AG	28	G = 0.06	
GG	2		
rs2306862			
CC	193	T = 0.86	0.336
CT	65	C = 0.14	
TT	3		

*p*<0.05 was considered statistically significant

**Table 3:** Association between *LRP5* SNPs and osteopenia/osteoporosis in studied population

SNPs/Genetic model/Genotype	Normal N (%)	Osteopenia/osteoporosis N (%)	OR (95% CI)	*p*-value
rs41494349				
Dominant model				
A/A	54 (90.0)	191 (88.8)	Reference	
A/G-G/G	6 (10.0)	24 (11.2)	1.13 (0.44-2.91)	0.800
Recessive model				
A/A-A/G	59 (98.3)	214 (99.5)	Reference	
G/G	1 (1.7)	1 (0.5)	0.28 (0.02-4.47)	0.380
rs2306862				
Dominant model				
C/C	39 (68.4)	154 (75.5)	Reference	
C/T-T/T	18 (31.6)	50 (24.5)	0.70 (0.37-1.34)	0.290
Recessive model				
C/C-C/T	56 (98.2)	202 (99.0)	Reference	
T/T	1 (1.8)	2 (1.0)	0.55 (0.05-6.23)	0.650

Note: rs3736228 was not analyzed due to none of its homozygous variant - TT genotype was detected

From Table 4, it can be observed that the wild-type group of rs3736228 was older than the risk allele group (CC vs CT, 58 vs 55, respectively, *p* = 0.002). However, BMI was comparable between these groups. From the various sites examined, the Mann–Whitney U test revealed a statistically significant difference between total radial and radial 33 BMD for rs3736228 and rs2306862. For rs3736228, the total radial BMD of the individuals or group carrying the risk allele (CT) was significantly higher than the wild-type group (CC), *p* = 0.011. In addition, the radial 33 BMD was higher in the individuals or group carrying the risk allele of rs3736228 (CT, *p* = 0.001) and rs2306862 (CT + TT, *p* = 0.015). However, there were no statistically significant differences between BMD values of the wild-type (AA) and risk (AG + GG) allele carrier group of the rs41494349 SNP.

**Table 4:** Association between *LRP5* genotypes and parameters of studied population

Characteristic	rs3736228		*p*-value	rs41494349		*p*-value	rs2306862		*p*-value
	CC	CT		AA	AG+GG		CC	CT+TT	
Age (years)	58 (45–73)	55 (45–75)	0.002	58 (45–75)	57 (45–72)	0.989	58 (45–73)	56 (45–75)	0.224
N	210	67		245	30		193	68	
BMI (kg/m^2^)	22.77 (15.46–39.54)	22.88 (18.75–33.91)	0.804	22.77 (15.46–39.54)	23.14 (19.34–30.47)	0.878	22.55 (15.46–39.54)	23.06 (18.44–33.91)	0.350
N	210	67		245	30		193	68	
Lumbar spine BMD (g/cm^2^)	1.018 (0.700–1.564)	0.983 (0.778–1.555)	0.584	1.013 (0.700–1.564)	1.003 (0.778–1.353)	0.388	1.013 (0.700–1.564)	0.993 (0.778–1.555)	0.787
N	160	45		183	22		147	46	
Femoral neck BMD (g/cm^2^)	0.787 (0.434–1.204)	0.789 (0.612–1.224)	0.728	0.785 (0.434–1.204)	0.831 (0.562–1.224)	0.154	0.785 (0.434–1.141)	0.788 (0.548–1.224)	0.905
N	178	55		210	23		165	56	
Total radial BMD (g/cm^2^)	0.469 (0.265–0.961)	0.500 (0.262–0.623)	0.011	0.475 (0.262–0.961)	0.475 (0.353–0.584)	0.702	0.471 (0.265–0.961)	0.485 (0.262–0.623)	0.191
N	210	67		245	30		193	68	
Radial 33 BMD (g/cm^2^)	0.614 (0.315–0.785)	0.657 (0.350–0.818)	0.001	0.623 (0.315–0.818)	0.634 (0.447–0.722)	0.769	0.613 (0.315–0.785)	0.649 (0.350–0.818)	0.015
N	210	67		245	30		193	68	
Total hip BMD (g/cm^2^)	0.870 (0.229–1.281)	0.875 (0.636–1.322)	0.565	0.870 (0.229–1.322)	0.909 (0.600–1.252)	0.625	0.869 (0.229–1.281)	0.876 (0.636–1.322)	0.636
N	178	55		210	23		165	56	

Data are presented as medians (min–max)

Because a greater radial 33 BMD was found in both rs3736228 and rs2306862 SNP risk allele carrier groups compared with their respective wild-type groups, it was of interest to determine whether a genotypic additive effect was present. Linkage disequilibrium (LD) analysis was conducted and a high level of LD was observed (D’/r^2^ = 0.9294/0.7549). Haplotype analysis was performed to observe the haplotypic effect of these two genotypes on causing osteopenia/osteoporosis using radial 33 BMD T-score cut-off. In both the normal and osteopenia/osteoporosis groups, CC haplotype was found at high frequency. The odds ratio of carrying the TT haplotype was found to be 0.48 (95 % CI = 0.26–0.88, *p* = 0.018) in comparison to the more common CC haplotype (Table 5). However, after adjusting for age, the TT haplotype was not found to be statistically significant: OR = 0.62 (95 % CI = 0.32–1.20, *p* = 0.160).

**Table 5:** Haplotype analysis: rs3736228 (C/T) and rs2306862 (C/T) SNP association using radial 33 BMD T-score cut-off

Haplotype^a^	Normal (Frequency)	Osteopenia/osteoporosis (Frequency)	OR	95% CI	*p*-value
CC	0.8331	0.8849	Reference haplotype		
TT	0.1418	0.0810	0.48	(0.26–0.88)	0.018
CT	0.0146	0.0298	1.85	(0.56–6.08)	0.310

*p*<0.05 was considered statistically significant

*OR* odds ratio, *95% CI* 95% confidence interval

^a^ Haplotype analysis was performed by SNPStat program [27]

**Fig. 1:**
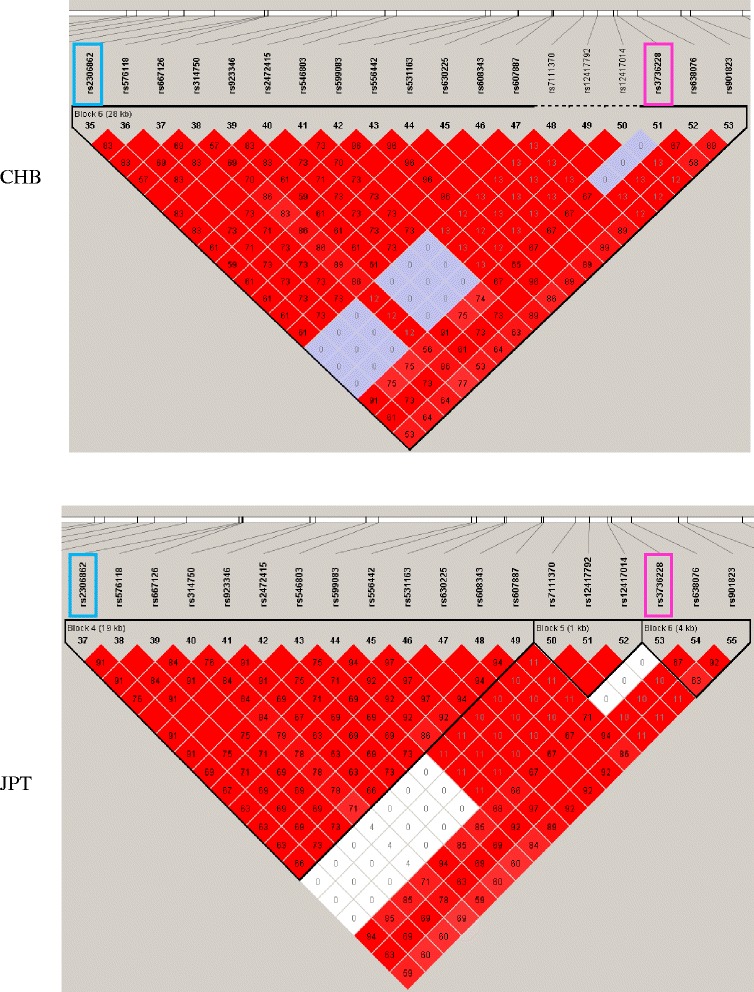
Linkage disequilibrium plot between *LRP5* SNPs showing region the rs3736228 and rs2306862 located using HapMap populations determined by Haploview 4.2 program. Each black triangle depicts haplotype blocks. LD is reported as D’. Bright red represents D’ = 1 and LOD ≥ 2, blue represents D’ = 1 and LOD < 2, pink represents D’ < 1, and LOD ≥ 2, and white represents D’ < 1 and LOD < 2. The *r* ^2^ values are shown in blocks. The rs3736228 and rs2306862 are indicated by boxes. CHB, Han Chinese in Beijing, China; JPT, Japanese in Tokyo, Japan

Although we did not detect an association between osteopenia/osteoporosis and haplotypic effect of the two SNPs, rs3736228 and rs2306862, they belonged to a big LD block comprising other variants in HapMap genotype data of Han Chinese in Beijing, China (CHB) and Japanese in Tokyo, Japan (JPT) population (Fig. 1). Haploview LD map of *LRP5* showed almost similar LD structure in both populations (Additional file 1 Figure S1). Both variants were in complete LD in CHB (block 6; D’/*r*^*2*^ = 1/0.913) and JPT despite located in non-adjacent blocks (block 4 and 6; D’/*r*^*2*^ = 1/0.941).

Univariate analysis was performed to determine if any factors contributed to osteoporosis, represented by total radial BMD T-score cut-off [27]. The factors tested were the rs3736228 genotype and those affecting osteoporosis risk, age and BMI. The combined effect of these factors was analyzed using binary logistic regression analysis (Table 6). For individuals with a BMI of less than 25 kg/m^2^, for every decade older, there was an increased risk of osteoporosis. The rs3736228 polymorphism had no effect.

**Table 6:** Logistic regression analysis of osteopenia/osteoporosis risk factors using total radial BMD T-score cut-off for osteoporosis

Factors	Crude OR	95% CI	*p*-value	Adjusted OR*	95% CI	*p*-value
rs3736228	2.67	0.91-7.88	0.075	2.43	0.74-7.99	0.144
BMI (≤25 kg/m^2^)	6.80	1.59-29.15	0.010	10.70	2.28-50.24	0.003
Age (increases every 10 years)	5.00	2.62-9.54	<0.001	6.32	3.05-13.07	<0.001

*BMD* bone mineral density, *OR* odds ratio, *95% CI* 95% confidence interval

## Discussion

*LRP5*, a co-receptor for the Wnt signaling pathway, is an important regulator for bone homeostasis [28]. Polymorphisms of *LRP5* have been demonstrated to have an influence on BMD and to be associated with an increased risk of osteoporosis. We investigated the effect of genetic variation of *LRP5* on BMD in Thai menopausal women.

Departure from HWE was detected in the rs3736228 genotype distribution. The most common cause of deviation from HWE is genotyping error [29]. However, for SNP it tends to be due to a deficit of heterozygotes, making allele dropout the most frequent cause of genotyping error [30]. On the contrary, with respect to our results however, it was likely due to a lack of homozygotes and the number of heterozygotes being higher than expected. This is in agreement with the Lewis phenotyping study showing that the cause of departure from HWE was a deficit of homozygotes [31].

In this study, we found that heterozygous risk allele carriers, the T-allele of rs3736228 (CT), had a significantly higher total radial BMD than those carrying homozygous C-allele carriers (CC), *p* = 0.011 (*N* = 210 vs 67, respectively). In addition, the risk allele carriers of rs3736228 and rs2306862 had a significantly higher radial 33 BMD than those with the wild-type allele (*p* = 0.001 (*N* = 210 vs 67) and *p* = 0.015 (*N* = 193 vs 68), respectively). However, the wild-type allele carrier group of rs3736228 was older than the risk allele group. Aging is an important confounding factor in osteoporosis, and an increase of age makes people more prone to developing osteoporosis, coinciding with a lower BMD. Therefore, it is likely that aging contributes to the lower radial BMD found in the wild-type group of rs3736228, as observed from logistic regression analysis. In postmenopausal women, age is reported to be the second most important predictor of BMD variation [32].

These findings are contradictory to previous studies in *LRP5* SNPs, whereby subjects with a risk allele have a lower BMD. The rs3736228 is associated with low BMD in various ethnic groups [18–21]. Furthermore, the association of rs2306862 and rs41494349 with BMD has been reported predominantly within the Asian population [23–25]. However, we failed to demonstrate the association of rs41494349 with BMD in our study.

The strong LD found between rs3736228 and rs2306862 is in agreement with a study by Mizuguchi et al [23]. In this study, the researchers identified an LD block between intron 7 and exon 18 of the *LRP5* gene in the Japanese women. This included rs2306862 on exon 10 and rs3736228 on exon 18. Haplotype blocks distribution generated by Haploview gave similar LD patterns between these two SNPs in CHB and JPT populations of HapMap. Although the TT haplotype showed a protective effect against osteopenia/osteoporosis, it disappeared after adjusting for age. This is likely because in the rs3736228 SNP, the wild-type allele carrier group was older than the risk allele group.

Our rs3736228 heterozygous risk allele carriers had a higher total radial BMD compared with the wild-type, which is in contrast to the majority of previous studies of *LRP5* polymorphisms, in which the rs3736228 risk allele carrier is associated with a reduced BMD [18–21]. However, our finding is consistent with two previous studies of rs3736228 in men. Kiel et al. showed that men with a reduced physical activity level carrying the risk allele of rs3736228 have a higher spine BMD, whereas Kruk et al found that men with no reported physical activity level have a high BMD at all hip sites [33, 34].

Both the Kiel and Kruk research groups suggest involvement of physical activity and mechanical loading in modulating the effect of *LRP5* on bone remodeling balance in response to physical exercise or on BMD determination. The mechanism by which the different alleles of rs3736228 polymorphism interact through physical activity to modify bone or BMD is unknown. However, *LRP5* and Wnt/beta-catenin signaling have been identified as essential components of mechanically induced signal transduction [35, 36]. Loading increases Wnt signaling pathway expression and Wnt/beta-catenin target genes; however, there is no response to mechanical loading in mice with deletion of *Lrp5* [37, 38].

Estrogen receptor alpha (ER-alpha) is involved in the bone adaptation process to mechanical loading [39]. Moreover, the *LRP5* and ER-alpha pathway are reported to interact during mechanical loading [40]. One study investigating gene-exercise interactions on BMD, showed an association between physical activity and ER-alpha polymorphism on BMD modulation at the loaded bone sites [41]. Furthermore, an interleukin-6-association study showed that its genetic variant influences cortical bone resorption during exercise [42]. Accordingly, it is possible that *LRP5* could have an effect on BMD in response to physical activity. Although no study has directly assessed the role of *LRP5* polymorphism, it remains to be determined if this polymorphism has an interaction with mechanical loading via physical activity or exercise, finally resulting in BMD change.

Our study has several limitations. First, because the DNA used for genotyping was derived from stored specimens, the sample size was fixed. Second, the available BMD data was much smaller in comparison with the genotyping data. Third, age is an important confounding factor for osteoporosis, which significantly different between the wild-type and risk allele carrier groups of rs3736228. Therefore, we cannot avoid or exclude the confounding factor effect of age. Fourth, our data deviated from HWE because of the higher incidence of heterozygotes found than expected. Taken together, this leads to difficulties in statistical analysis; giving rise to false positives or negatives and even non-significant results. Lastly, other potential confounders, such as years since menopause, physical activity, smoking, alcohol consumption and others, were not assessed.

## Conclusions

From our findings and limitations, it seems that *LRP5* polymorphisms are not a risk factor for osteoporosis in Thai menopausal women. However, this research is not conclusive and further studies with a larger sample size, and that address all the aforementioned limitations, will help clarify the role of *LRP5* as a genetic determinant of osteoporosis.

## Methods

### Study population

The original research project was the collaboration between the Department of Obstetrics and Gynecology, Faculty of Medicine, Ramathibodi hospital and the Department of Tropical Nutrition and Food Science, Faculty of Tropical Medicine with EC approval from Ramathibodi hospital since 2009. Data and blood collection was performed at the Ramathibodi hospital and the experiments were performed at the Faculty of Tropical Medicine. In the present study, we used stored specimen left from that original project with permission and EC approval.

In this study, subjects included 277 menopausal Thai women who attended the menopause post-operation follow-up clinic at the Department of Obstetrics and Gynecology, Ramathibodi Hospital, Bangkok, Thailand. Inclusion criteria used were: (1) women with 45 years of age or older; (2) not having serious disease history; (3) not receiving vitamin/mineral for bone supplement; and (4) willing to participate in the study. All subjects were in good health and gave informed consent to participate in the study. Exclusion criteria included any known diseases that could affect bone metabolism such as hyperthyroidism, hyperparathyroidism, rheumatoid arthritis, rickets and osteomalacia, hypogonadism, type I diabetes mellitus, and Cushing’s syndrome. Physical examinations were conducted by the same physician. The study protocol was approved by the Ethics committees of the Faculty of Tropical Medicine and Faculty of Medicine (Ramathibodi Hospital), Mahidol University, Bangkok, Thailand.

### BMD measurement

Bone mineral density (BMD, g/cm^2^) was assessed at the lumbar spine (L2–L4), femoral neck, total radius, radial 33, and total hip using dual-energy X-ray absorptiometry (DEXA) (Lumar Prodigy, Lunar, USA) by a single, experienced technician.

### DNA extraction and genotyping

Genomic DNA was extracted from EDTA-treated peripheral blood samples using the Flexi Gene DNA kit (Qiagen, Hilden, Germany). Genomic DNA was stored at −70 °C for further analysis. Genotyping of *LRP5* SNP variation was conducted using the polymerase chain reaction-restriction fragment length polymorphism (PCR-RFLP) method.

The forward and reverse primers for A1330V (rs3736228) SNP were designed as 5′-GACTGTCAGGACCGCTCACACG-3′ and 5′-AAGGTTTTCAGAGCCCCTAC-3′, respectively. The PCR product was cut by *Dra*III (New England Biolabs, Beverly, CA) and separated by 6 % polyacrylamide gel electrophoresis. DNA from a subject homozygous for the C allele appeared as a band of 143 bp in length relative to the 100 bp size marker. The T allele constructs cut site for *Dra*III, and DNA from a subject homozygous for the T allele, appeared as bands of 119 and 24 bp, respectively [25].

The forward and reverse primers for Q89R (rs41494349) SNP were designed as 5′-CTCTGGGCATAGTGCTCCATC-3′ and 5′-CCGGAGATGACCACGTTCTG-3′, respectively. The PCR product was cut by *Ava*I (New England Biolabs, Beverly, CA) and separated by 6 % polyacrylamide gel electrophoresis. DNA from a subject homozygous for the A allele appeared as a band of 308 bp in length relative to the 100 bp size marker. The G allele constructs cut site for *Ava*I, and DNA from a subject homozygous for the G allele, appeared as bands of 257 and 51 bp, respectively.

The forward and reverse primers for N740N (rs2306862) SNP were designed as 5′-CTACTGGGCCGACACTGGGATTAA-3′ and 5′-ACAGCTCTAATCACCGAGGG-3′, respectively. The PCR product was cut by *Ase*I (New England Biolabs, Beverly, CA) and separated by 6 % polyacrylamide gel electrophoresis. DNA from a subject homozygous for the C allele appeared as a band of 237 bp in length relative to the 100 bp size marker. The T allele constructs cut site for *Ase*I, and DNA from a subject homozygous for the T allele appeared as bands of 216 and 21 bp, respectively [25].

**Fig. 2:**
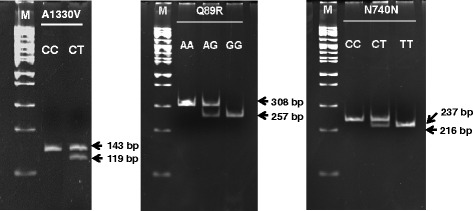
Fragment patterns of *LRP5* SNPs with a 100 bp marker. M, Marker

Results have been either randomly duplicated checked and performed direct sequencing. Resolution of 24 bp (A1330V), 51 bp (Q89R) and 21 bp (N740N) cleaved products cannot be resolved in 6 % PAGE and are invisible on the gel. Therefore, only DNA bands with larger size for each SNP can be seen. Gel depicted 3 SNPs genotyping is shown below in Fig. 2.

### Statistical analysis

The SPSS v.15.0 program for Windows (SPSS, Chicago, IL) was used to analyze the median, range and *p* value (Mann–Whitney U test) between groups. Departure from Hardy–Weinberg Equilibrium (HWE) of allele frequency for each SNP was tested by the Chi-square test. Linkage disequilibrium (LD) and haplotype analysis were performed using the SNPStat web tool program [43]. LD plot of CHB and JPT populations genotype data from HapMap phase 3 (June 15, 2016) was generated using Haploview 4.2 program [44].

Univariate analysis was performed to determine any factor that had an effect on osteoporosis, represented by total radial BMD T-score cut-off for osteoporosis. Any variable with *p* ≤ 0.1 in univariate analysis was further analyzed in stepwise multivariate logistic regression analysis using a backward Wald method for determining independent associated factors for osteoporosis. Backward Wald method is a stepwise entry method used to construct the logistic regression model by removing explanatory variables from the full model that including all the specified explanatory variables. All tests of significance were 2-sided, and a *p*-value <0.05 was considered statistically significant.

## Additional file

Additional file 1: Haplotype blocks distribution in the LRP5 gene of CHB and JPT populations of HapMap generated by Haploview 4.2 program. Each black triangle depicts haplotype blocks. LD is reported as D′. Bright red represents D' = 1 and LOD ≥ 2, blue represents D' = 1 and LOD < 2, pink represents D' < 1, and LOD ≥ 2, and white represents D' < 1 and LOD < 2. The r2 values are shown in blocks. CHB, Han Chinese in Beijing, China; JPT, Japanese in Tokyo, Japan.
