# Supplementary material for: Low density lipoprotein receptor-related protein 5 gene polymorphisms and osteoporosis in Thai menopausal women
Source: J Negat Results Biomed. 2016 Sep 1;15(1):16. doi: 10.1186/s12952-016-0059-7 (PMC5007848; doi:10.1186/s12952-016-0059-7)
Supplement: Additional file 1: — Haplotype blocks distribution in the LRP5 gene of CHB and JPT populations of HapMap generated by Haploview 4.2 program. Each black triangle depicts haplotype blocks. LD is reported as D′. Bright red represents D' = 1 and LOD ≥ 2, blue represents D' = 1 and LOD < 2, pink represents D' < 1, and LOD ≥ 2, and white represents D' < 1 and LOD < 2. The r2 values are shown in blocks. CHB, Han Chinese in Beijing, China; JPT, Japanese in Tokyo, Japan. [file 12952_2016_59_MOESM1_ESM.docx]

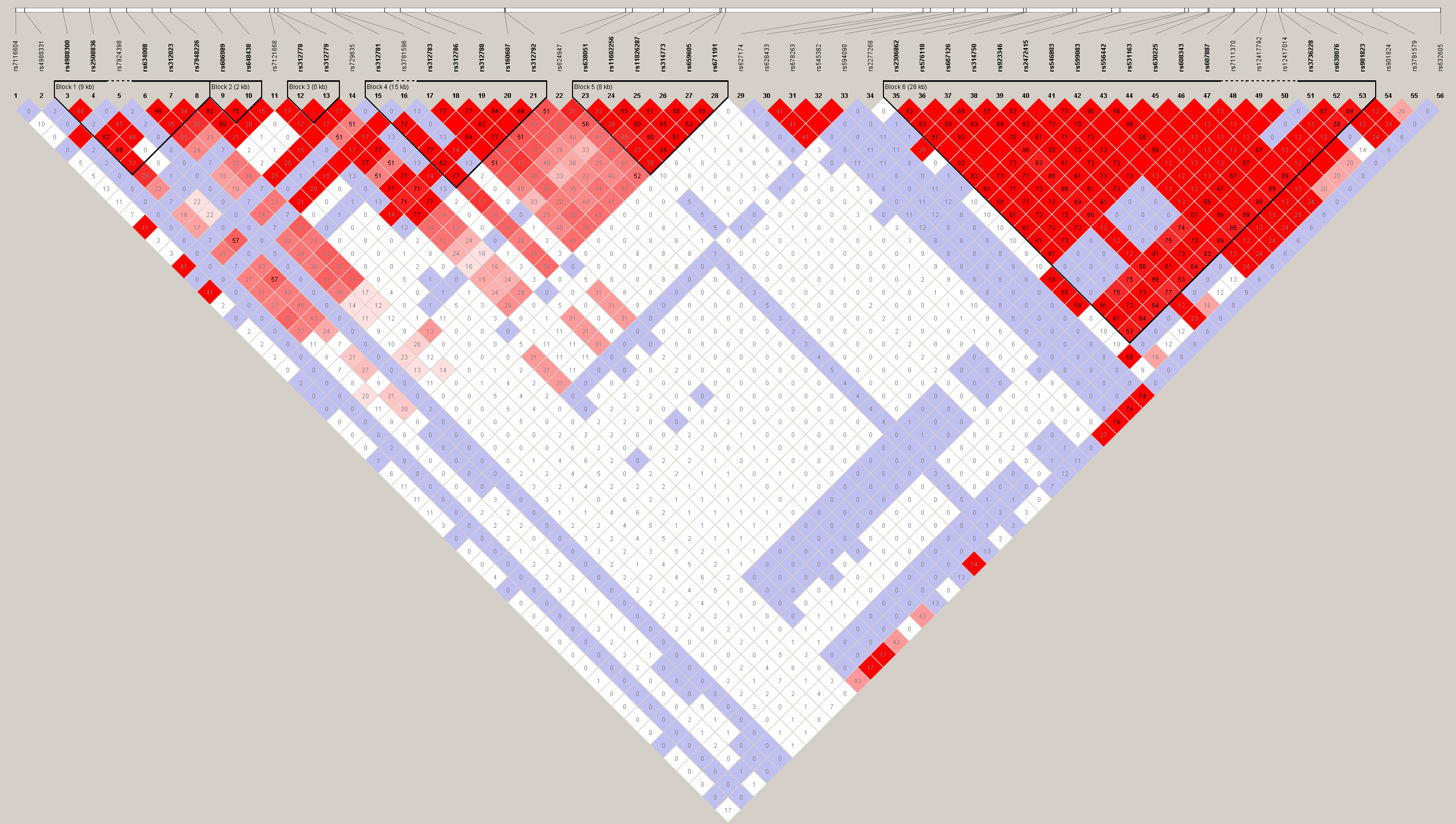


CHB


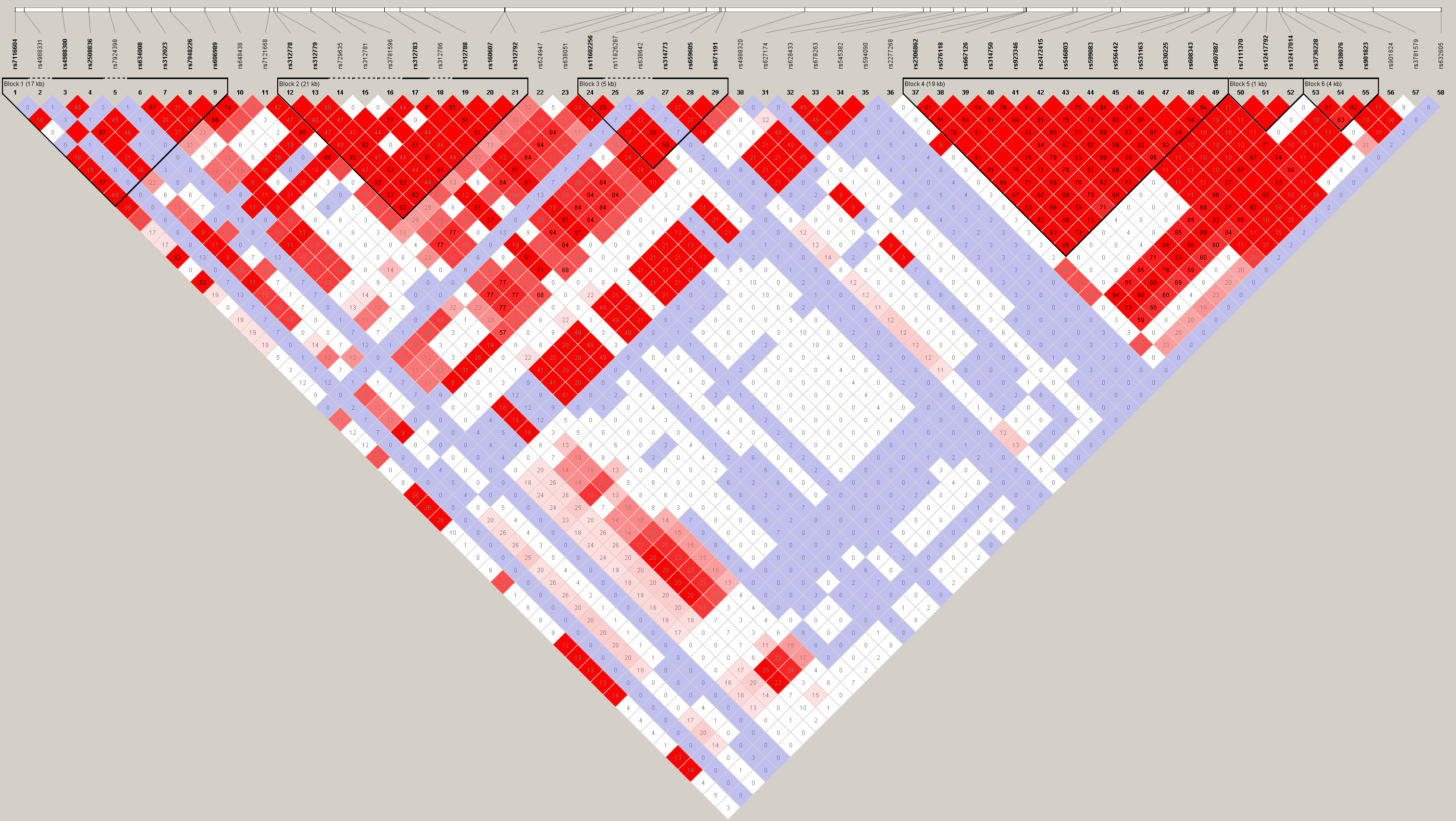


JPT

**Supplementary Figure 1** Haplotype blocks distribution in the *LRP5* gene of CHB and JPT populations of HapMap generated by Haploview 4.2 program. Each black triangle depicts haplotype blocks. LD is reported as D′. Bright red represents D' = 1 and LOD ≥ 2, blue represents D' = 1 and LOD < 2, pink represents D' < 1, and LOD ≥ 2, and white represents D' < 1 and LOD < 2. The *r*^2^ values are shown in blocks. CHB, Han Chinese in Beijing, China; JPT, Japanese in Tokyo, Japan
